# Associations between body mass index and peak expiratory flow rate and the mediating role of hand grip strength: evidence from CHARLS

**DOI:** 10.1016/j.clinsp.2026.101091

**Published:** 2026-09-11

**Authors:** Xiaonan Zhang, Haowei Jin, Sheng Zhou

**Affiliations:** The First Affiliated Hospital of Anhui Medical University, Anhui, China

**Keywords:** Middle-aged and elderly, Peak expiratory flow, Body mass index, Grip strength, Mediation analysis, Lung function

## Abstract

**Objective:**

To investigate the mediating role of Hand Grip Strength (HGS) in the relationship between Body Mass Index (BMI) and Peak Expiratory Flow (PEF) among Chinese middle-aged and elderly adults without diagnosed lung disease.

**Method:**

This cross-sectional study utilized data from the 2015 China Health and Retirement Longitudinal Study (CHARLS). HGS, BMI, and PEF were assessed through standardized measurements. Logistic regression models were applied to evaluate the associations of BMI and HGS with reduced PEF, defined as PEF%pred < 80%. Mediation analysis was conducted to explore the relation between BMI and PEF%pred via HGS.

**Result:**

A total of 8784 participants were included in the analysis, with 671 classified as having reduced PEF (PEF%pred < 80%). Higher BMI and greater HGS were each associated with significantly lower odds of reduced PEF (per 1-unit increase in BMI: OR = 0.970, 95% CI: 0.946–0.993; per 1 kg increase in HGS: OR = 0.961, 95% CI: 0.952–0.970), implying that lower BMI and lower HGS are related to increased risk. Mediation analysis indicated that HGS statistically mediated the association between BMI and PEF%pred; however, the absolute indirect effect size was very small (indirect effect = 0.003, 95% CI: 0.003–0.004), corresponding to a negligible change in PEF%pred.

**Conclusion:**

Both low BMI and reduced grip strength are significantly associated with greater odds of reduced PEF in Chinese middle-aged and elderly adults. Although HGS mediates part of the statistical association between BMI and PEF, the clinical significance is limited due to the small effect size. These results do not support specific muscle-strengthening interventions based on this finding alone, and caution should be exercised in clinical interpretation.

## Highlights

• BMI and HGS are negatively associated with reduced PEF in Chinese middle-aged and elderly.
• HGS partially mediates BMI–PEF association in a large nationally representative sample.
• Mediating effect is small and does not support targeted muscle-strengthening interventions.
• First study to identify HGS as a mediator between BMI and PEF%pred in non-lung-disease adults.

## Introduction

Chronic respiratory diseases, including Chronic Obstructive Pulmonary Disease (COPD), asthma, and restrictive ventilatory dysfunction ‒ represent a significant and growing public health challenge among middle-aged and older adults^1^^,^^2^ Globally, COPD alone affects over 250 million people and is now the third leading cause of death^3^ In China, approximately 13.7% of adults aged 40-years and above are affected by chronic respiratory diseases, with risk increasing substantially with age^4^ Notably, even in individuals without diagnosed lung disease, subclinical respiratory impairment is prevalent and is associated with increased risks of cardiovascular events and mortality^5^ As such, routine monitoring of respiratory function is essential for early detection and intervention to improve quality of life in this vulnerable population. Peak Expiratory Flow (PEF) ‒ the maximum flow rate achieved during forced expiration ‒ offers a simple, cost-effective, and reliable means of assessing respiratory function^6^^,^^7^ PEF is widely used in population-level respiratory health assessments and disease screening, serving not only as a marker for reduced PEF but also as a predictor of pneumonia and mortality risk in older adults^8^, ^9^, ^10^

Preserving pulmonary function is critical for healthy aging. However, the determinants of declining respiratory function among community-dwelling, non-diseased older adults remain incompletely understood. Both lower and higher Body Mass Index (BMI) have been independently associated with impaired respiratory function in middle-aged and elderly adults. Specifically, lower BMI is linked to reduced lung volumes and airflow, while higher BMI may impair respiratory mechanics due to excess adiposity^11^ Muscle strength, as reflected by Hand Grip Strength (HGS), has also been associated with pulmonary function indicators such as PEF and forced expiratory volume, suggesting that muscle weakness may contribute to reduced ventilatory capacity^12^^,^^13^ Notably, recent evidence has highlighted that muscle strength may partially mediate the influence of nutritional status or body composition on pulmonary function ‒ especially through the role of respiratory muscles such as the diaphragm, which are essential for generating and sustaining adequate expiratory flow^14^^,^^15^ Despite these findings, the specific mediating role of muscle strength in the relationship between BMI and respiratory function in healthy, community-dwelling elderly adults remains largely unexplored, particularly in China’s aging population. It is hypothesized that the association between BMI and respiratory function is mediated by systemic muscle strength. In older adults, a lower BMI often reflects sarcopenia and reduced metabolic reserve, rather than simply the absence of obesity-related comorbidities. Handgrip strength, a validated marker of overall muscle function, is used in this study, which utilizes data from the 2015 wave of the China Health and Retirement Longitudinal Study (CHARLS)^16^ The study aims to investigate the mediating role of muscle strength in the BMI-respiratory function relationship in community-dwelling older adults without diagnosed lung disease, providing evidence to inform prevention and intervention strategies for reduced PEF in this population.

## Methods

### Study design and population

The CHARLS is a nationwide, prospective cohort study designed to collect a high-quality, nationally representative sample of Chinese residents aged 45-years and older for research on aging (http://CHARLS.pku.edu.cn/en). The baseline national wave began in 2011, including about 10,000 households and 17,500 individuals from 150 counties/districts and 450 villages/resident committees. For this analysis, we used data from the 2015 wave.

**Fig. 1:**
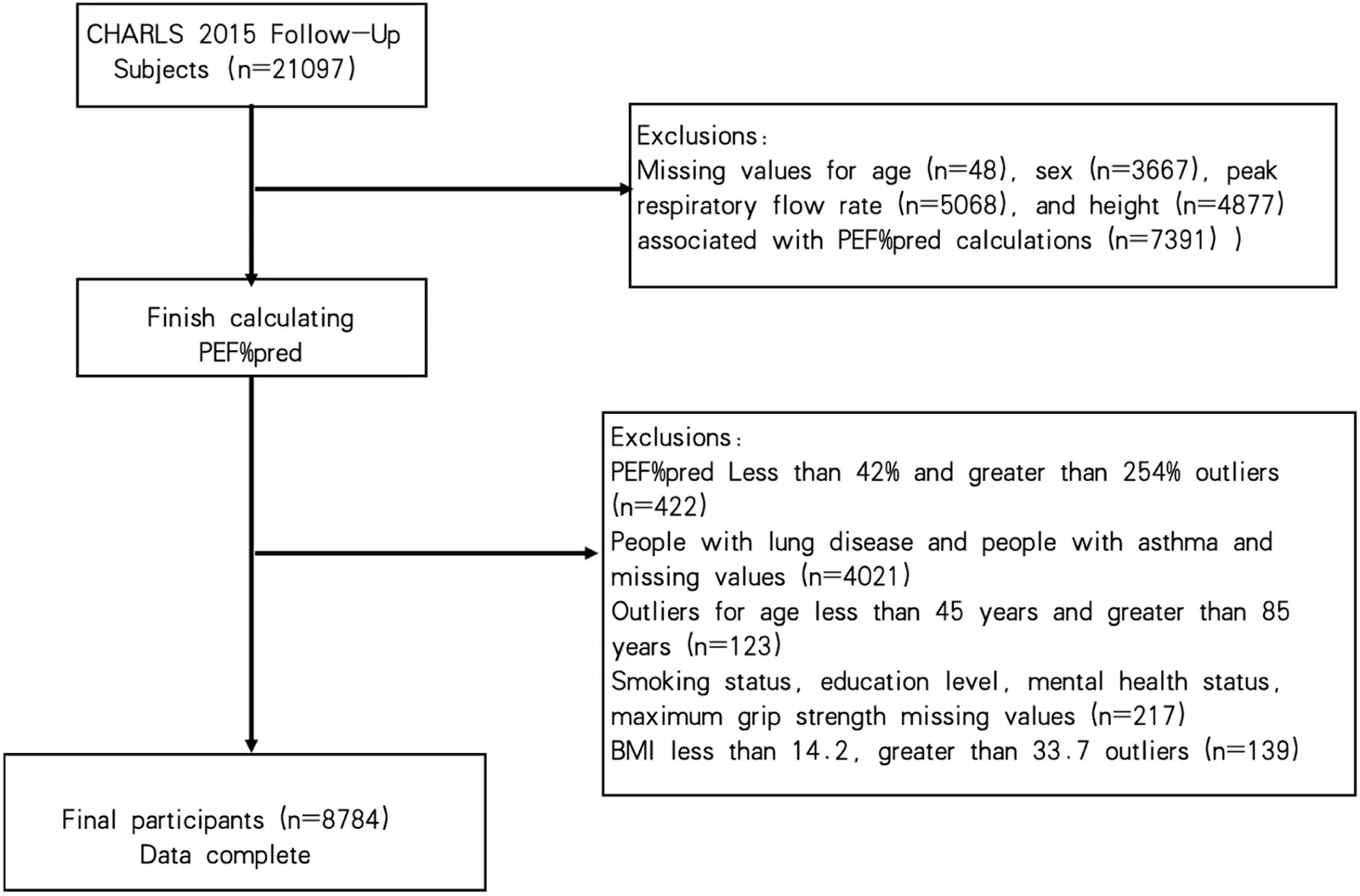
Flowchart of the study design.

Participants with missing values for height, age, sex, or PEF were excluded. Individuals with self-reported lung disease or asthma were also omitted to focus on a non-diseased population. Outliers in PEF%pred, age, and BMI were addressed using box plots, retaining only those with PEF%pred between 42% and 254%, aged 45–85 years, and BMI between 14.2 and 33.7. Further exclusions were made for missing data on smoking status, education level, mental health status, and grip strength. As a result of these strict criteria, over 12,000 cases were excluded; this approach was necessary to ensure data quality and minimize potential confounding.Detailed processing information is presented in Fig. 1. To assess potential selection bias, we compared key baseline characteristics between the final included cohort (n n = 8784) and participants excluded solely due to missing data (n = 7192). No significant differences were observed in age and BMI between the two groups. Although sex distribution differed significantly (p < 0.01), this discrepancy was mainly caused by additional exclusion of participants with pulmonary diseases and asthma during further screening, which affected male participants to a greater extent. Collectively, the baseline characteristics were generally comparable between the two groups, indicating no obvious intentional selection bias in participant enrollment. All respondents provided informed consent, and the CHARLS protocol was approved by the Biomedical Ethics Committee of Peking University (IRB00001052-11015). The Ethics Committee of Guang’anmen Hospital of the China Academy of Traditional Chinese Medicine exempted secondary analysis of this database from further ethical review.

### Assessment of peak expiratory flow (PEF)

PEF was measured using a peak flow meter (Everpure™, Shanghai, China). Participants were instructed to take a deep breath, seal their lips around the mouthpiece, and exhale forcefully and rapidly. The procedure was performed three times at 30-second intervals, with the highest value recorded for analysis. The predicted PEF was calculated using equations validated by Nanshan Zhong: for men, $\begin{array}{c} 75.6+20.4\times age-0.41\times age^{2}+0.002\times age^{3}+1.19\times height;\;for\;woman,\\ 282.0+1.79\times age-0.046\times age^{2}+0.68\times height \end{array}$. In these equations, age is expressed in years, and height in centimeters. A threshold of PEF%pred < 80% was used, based on conventional clinical practice and recent literature^14^ We acknowledge that this cut-off is somewhat arbitrary and has not been universally validated for PEF in community-dwelling populations; in this study it is used primarily for analytical convenience to define a group with relatively lower expiratory performance, while PEF%pred itself is best interpreted as a continuous measure of expiratory function rather than a diagnostic threshold. Given the limitations of PEF as a standalone measure ‒ particularly its effort-dependence and insensitivity to small airway or restrictive defects ‒ this outcome is referred to as “reduced PEF” rather than “airflow limitation”.

### Measurement of hand grip strength (HGS)

HGS was measured using the Nantong WCS-100 dynamometer. Participants gripped the device as tightly as possible, elbows at right angles, and maintained the grip for a few seconds. Two readings were taken for each hand; the maximum value among the four measurements was used for analysis. Grip strength is widely recognized as a clinically meaningful indicator of low muscle strength and sarcopenia^15^^,^^17^^,^^18^

### Calculation and classification of body mass index (BMI)

BMI was calculated as weight (kg) divided by height squared (m^2^) using 2015 measurements. BMI categories followed both Chinese and World Health Organization (WHO) criteria: underweight (< 18.5), normal weight (18.5–24), overweight (24–28), and obesity (≥ 28)^19^

### Covariates

Covariates included age (continuous), sex (male/female), smoking status (yes/no), education level (primary, elementary, middle school, high school or above), and depression status (yes/no, defined as CESD-10 score ≥ 10)^6^

### Statistical methods

All statistical analyses were performed using R software (version 4.3.0), with a significance level set at α = 0.05. The Shapiro-Wilk test was used to assess normality of continuous variables. Baseline characteristics were summarized as mean ± SD for normally distributed variables and median (IQR) for skewed variables; categorical variables as n (%). Comparisons used *t*-tests or ANOVA for continuous variables and Chi-Square tests for categorical variables, as appropriate. For non-normally distributed variables such as BMI and HGS, medians and IQRs are reported, and these variables were also analyzed as continuous predictors in restricted cubic spline and mediation models to assess dose-response and indirect effects.

To address confounding, a series of logistic regression models were constructed: Model A (unadjusted); Model B (adjusted for age and sex); Model C (additionally adjusted for education, smoking, depression); Model D (further adjusted for chronic diseases). The mediating effect of HGS on the BMI–PEF%pred association was examined using the PROCESS macro for mediation analysis (Andrew Hayes). Bootstrap resampling (10,000 iterations) provided 95% Confidence Intervals for indirect effects. If the bootstrap CI excluded zero, mediation was considered statistically significant. In all models, BMI was the independent variable, HGS the mediator, and PEF%pred the dependent variable. Because the total effect of BMI on PEF%pred was very small in absolute terms, our primary focus was on reporting the unstandardized indirect effect and its 95% confidence interval, rather than the proportion of the total effect mediated. The proportion mediated was calculated for completeness but is presented only in the Supplementary Material to avoid over-interpreting a large percentage applied to a negligible effect size. To address concerns about cut-off selection and potential misclassification, sensitivity analyses were performed using alternative PEF%pred thresholds (70%, 90%, and 100%). These analyses were conducted to assess the robustness of our results across different threshold settings. The STROBE Statement were followed throughout the entire cross-sectional study.

## Results

### Baseline characteristics

A total of 8784 participants were included in the analysis, with 671 (7.6%) classified as having reduced PEF (PEF%pred < 80%) and 8113 (92.4%) as having PEF%pred ≥80%. The median age of participants was 60-years (IQR: 53–66), with 57.1% females and 42.9% males. The median BMI was 23.8 kg/m^2^ (IQR: 21.4–26.2), and the median Hand Grip Strength (HGS) was 29.0 kg (IQR: 23.0–37.0). Participants with reduced PEF had lower BMI and HGS compared to those with higher PEF%pred values. They also had higher CESD-10 scores, indicating worse mental health status. Significant group differences were observed in gender distribution, smoking status, and education level (all p < 0.05). No significant differences were found for comorbidities such as hypertension, diabetes, heart disease, gastrosis, or nephrosis (all p > 0.05). Detailed baseline characteristics are summarized in Table 1.

**Table 1:** Baseline characteristics of study participants.

Variable	Total (= 8784)	PEF%pred ≥ 80% (n = 8113)	PEF%pred < 80% (n = 671)	p-value
Gender				<0.001
Female	5015 (57.1%)	4529 (55.8%)	486 (72.4%)	
Male	3769 (42.9%)	3584 (44.2%)	185 (27.6%)	
Smoking				0.027
No	6470 (73.7%)	5951 (73.4%)	519 (77.4%)	
Yes	2314 (26.3%)	2162 (26.7%)	152 (22.7%)	
Age (years)	60 (53, 66)	60 (53, 66)	60 (52, 66)	0.404
Education				<0.001
Below elementary school	3882 (44.2%)	3503 (43.2%)	379 (56.5%)	
Elementary school	1967 (22.4%)	1813 (22.4%)	154 (23.0%)	
Middle school	1952 (22.2%)	1845 (22.7%)	107 (16.0%)	
High school and above	983 (11.2%)	952 (11.7%)	31 (4.6%)	
HGS (kg)	29.0 (23.0, 37.0)	29.6 (23.5, 37.5)	24.5 (19.0, 31.1)	<0.001
CESD-10	6.0 (3.0, 11.0)	6.0 (3.0, 11.0)	7.0 (3.0, 12.0)	<0.001
BMI (kg/m²)	23.8 (21.4, 26.2)	23.8 (21.5, 26.3)	23.2 (20.9, 25.9)	0.001
Hypertension				0.818
Yes	2651 (30.6%)	2446 (30.5%)	205 (31.1%)	
No	6016 (69.4%)	5561 (69.5%)	455 (68.9%)	
Diabetes				0.460
Yes	782 (9.0%)	728 (9.1%)	54 (8.2%)	
No	7885 (91.0%)	7277 (90.9%)	608 (91.8%)	
Heart disease				0.064
Yes	1239 (14.2%)	1128 (14.0%)	111 (16.7%)	
No	7469 (85.8%)	6916 (86.0%)	553 (83.3%)	
Nephrosis				0.338
Yes	703 (8.1%)	642 (8.0%)	61 (9.1%)	
No	8017 (91.9%)	7408 (92.0%)	609 (90.9%)	
Gastrosis				0.077
Yes	2647 (30.4%)	2425 (30.1%)	222 (33.5%)	
No	6069 (69.6%)	5628 (69.9%)	441 (66.5%)	

Note: Values are presented as n (%) for categorical variables and median (interquartile range) for continuous variables due to non-normal distribution. Continuous variables were compared using the Mann-Whitney *U*-test; categorical variables were compared using the chi-square test. HGS, Hand Grip Strength (maximum grip strength of both hands); CESD-10, Center for Epidemiologic Studies Depression Scale (scores > 10 suggest depression); BMI, Body Mass Index. Reduced PEF as PEF%pred < 80%.

### Association of BMI and HGS with risk of reduced PEF

Multivariable logistic regression analyses were performed to examine the association between BMI, HGS, and the odds of reduced PEF, with results summarized in Table 2, Table 3. Low BMI and reduced grip strength are significantly associated with reduced PEF. In the fully adjusted model (Model D, Table 3), each one-unit decrease in HGS was associated with a 3.9% increase in the odds of reduced PEF (OR = 0.961, 95% CI: 0.952–0.970, p < 0.001), and each one-unit decrease in BMI was associated with a 3.0% increase in the odds (OR = 0.970, 95% CI: 0.945–0.995, p = 0.021). These associations remained stable across all models with progressive adjustment for demographic, socioeconomic, and clinical variables.

**Table 2:** Potential variables associated with the reduced PEF.

Characteristic	OR	95% CI	p-value
HGS (per 1 kg ↓)	0.961	0.952, 0.970	<0.001
BMI (per 1 unit ↓)	0.970	0.946, 0.993	0.013
Age (per 1 year ↑)	0.974	0.964, 0.984	<0.001
Gender			
Female	Reference		
Male	0.699	0.543, 0.895	0.005
Education			
Below elementary school	Reference		
Elementary School	0.904	0.736, 1.106	0.332
Middle School	0.662	0.520, 0.837	<0.001
High school and above	0.403	0.269, 0.583	<0.001
Smoking			
No	Reference		
Yes	1.438	1.123, 1.839	0.004

Note: Odds Ratios (OR) and 95% Confidence Intervals (95% CI) were estimated using logistic regression. The dependent variable is reduced PEF (PEF%pred < 80%). For continuous variables, the OR reflects the change in odds per unit decrease (for HGS and BMI) or increase (for age). For example, for HGS, each 1 kg increase is associated with a 3.9% decrease in the odds of reduced PEF (OR = 0.961), implying that higher muscle strength is a protective factor against reduced expiratory performance.

**Table 3:** Associations of Body Mass Index (BMI) and Hand Grip Strength (HGS) with odds of Suboptimal Expiratory Function (PEF%pred < 80%) in stepwise logistic regression models.

Variable	Model A	Model B	Model C	Model D
	OR (95% CI), p	OR (95% CI), p	OR (95% CI), p	OR (95% CI), p
HGS	0.957 (0.950, 0.964), < 0.001	0.959 (0.950, 0.967), < 0.001	0.961 (0.952, 0.970), < 0.001	0.961 (0.952, 0.970), < 0.001
BMI	0.973 (0.950, 0.996), 0.022	0.961 (0.938, 0.984), 0.001	0.970 (0.946, 0.993), 0.013	0.970 (0.945, 0.995), 0.021
Age (per 1 year ↑)		0.980 (0.970, 0.990), < 0.001	0.974 (0.964, 0.984), < 0.001	0.972 (0.961, 0.982), < 0.001
Gender				
Female	Reference	Reference	Reference	Reference
Male		0.772 (0.629, 0.944), 0.012	0.699 (0.543, 0.895), 0.005	0.701 (0.541, 0.904), 0.007
Education				
Below elementary school	Reference	Reference	Reference	Reference
Elementary School			0.904 (0.736, 1.106), 0.332	0.899 (0.727, 1.106), 0.317
Middle School			0.662 (0.520, 0.837), < 0.001	0.667 (0.521, 0.849), 0.001
High school and above			0.403 (0.269, 0.583), < 0.001	0.390 (0.256, 0.571), < 0.001
Smoking				
No	Reference	Reference	Reference	Reference
Yes			1.438 (1.123, 1.839), 0.004	1.468 (1.141,1.886), 0.003
CESD-10 (Depression)				
No Depression				Reference
Depression				1.074 (0.901, 1.277), 0.423
Hypertension				1.059 (0.873, 1.282), 0.557
Diabetes				0.881 (0.643, 1.184), 0.415
Heart disease				1.270 (1.001, 1.598), 0.045
Nephrosis				1.118 (0.827, 1.487), 0.454
Gastrosis				0.981 (0.819, 1.171), 0.832

Note: Odds Ratios (OR) and 95% Confidence Intervals (95% CI) are presented for four logistic regression models. Model A: Unadjusted; Model B: Age + Sex Adjusted; Model C: Age + Sex + Education + Smoking; Model D: Adjusted for age, sex, education, smoking, CESD-10, hypertension, diabetes, heart disease, nephrosis, and gastrosis.

For continuous variables, the OR reflects the change in odds per unit increase (for HGS, BMI, and age). For example, each 1 kg increase in HGS is associated with a 3.9% reduction in the odds of suboptimal expiratory function (OR = 0.961), and each 1-unit increase in BMI is associated with a 3.0% reduction (OR = 0.970).

**Fig. 2:**
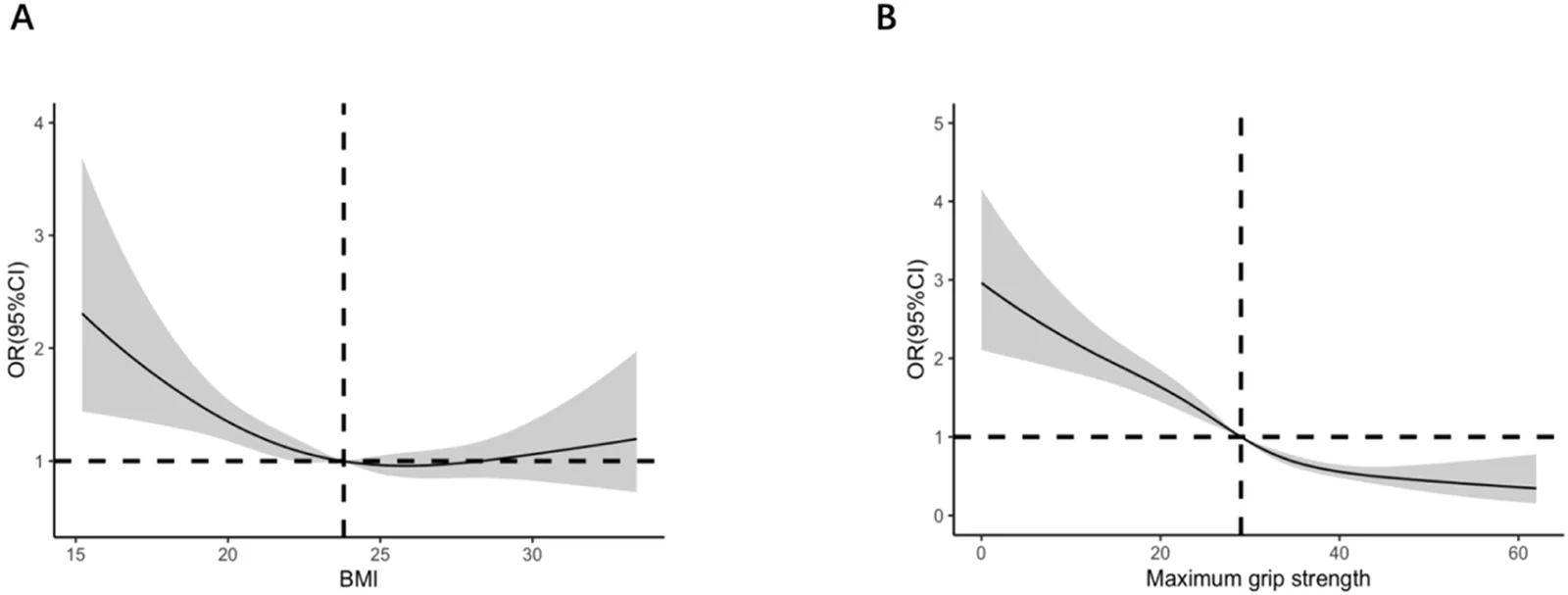
Restricted cubic spline analysis. (A) The association between BMI and the risk of reduced PEF. (B) The association between HGS and the risk of reduced PEF. Both plots show a non-linear, inverse relationship: lower BMI and lower HGS are associated with a greater risk of reduced PEF, while higher values correspond to lower risk. Shaded areas represent 95% confidence intervals. Analyses are adjusted for age, sex, and relevant covariates.

Restricted cubic spline analyses further illustrated that the odds of reduced PEF increased as BMI and HGS decreased, with the odds ratio crossing 1 at approximately 23.8 kg/m^2^ for BMI and 29 kg for HGS. The association became more pronounced below these values (Fig. 2).

Other demographic and lifestyle factors were also independently associated with reduced PEF. Older age was associated with lower odds (OR = 0.972, p < 0.001), while male gender was associated with lower odds compared to female (OR = 0.701, p = 0.007). Higher educational attainment demonstrated a graded inverse relationship with risk; individuals with a high school education or above had 61% lower odds (OR = 0.390, p < 0.001) compared to those with less than elementary education. Current smoking status was associated with higher odds (OR = 1.468, p = 0.003). Among comorbidities included in the final model, only a history of heart disease was significantly associated with increased odds (OR = 1.270, p = 0.045). Depression, hypertension, diabetes, nephrosis, and gastrosis were not significantly associated with the outcome in this sample. For BMI, each 1-unit decrease corresponded to approximately a 3.1% increase in the odds of reduced PEF (1/0.970 ≈ 1.031).

### Mediating role of HGS in the association between BMI and PEF%pred

To explore potential pathways linking BMI and PEF%pred, we examined the mediating role of HGS using mediation analysis. BMI was positively associated with HGS (β = 0.302, SE = 0.033, p < 0.001). When both BMI and HGS were included in the model for PEF%pred, the direct association between BMI and PEF%pred was not statistically significant (β = 0.001, SE = 0.001, p > 0.05), while HGS remained significantly associated with PEF%pred (β = 0.011, SE < 0.001, p < 0.001) (Table 4).

**Table 4:** Mediating effect of HGS on the relationship between BMI and PEF%pred.

	HGS			PEF%pred		
	Model 1		Model 2		Model 3	
	β	SE	β	SE	β	SE
**BMI**	0.302^c^	0.033	0.004^b^	0.001	0.001	0.001
**HGS**	‒	‒	‒	‒	0.011^c^	0.001
**R²**	0.009				0.001	
**F**	82.07^c^				9.16^b^	

Notes: Model 1: Effect of BMI on the mediator (HGS). Model 2: Effect of BMI on PEF%pred without considering the mediator. Model 3: Effect of BMI and HGS on PEF%pred (with HGS as mediator). All models adjusted for relevant covariates (n = 8784).

β, Regression coefficient; SE, Standard Error.

Significance: * p < 0.05.

b p < 0.01.

c p < 0.001.

Bootstrapping with 10,000 replicates showed a statistically significant total association between BMI and PEF%pred (effect size = 0.004, SE = 0.001, 95% CI: 0.001–0.007), but the direct effect was not significant (effect size = 0.001, SE = 0.001, 95% CI: −0.002 to 0.003). The indirect association of BMI with PEF%pred via HGS was statistically significant (effect size = 0.003, SE < 0.001, 95% CI: 0.003–0.004), with a fully standardized indirect effect of 0.026 (2.6%) (SE = 0.003, 95% CI: 0.020–0.032) (Table 5).

**Table 5:** Effect of HGS on the Relationship Between BMI and PEF%pred.

Effect	Effect Size	Standard Error	95% Confidence Interval
Total effect (direct + indirect)	0.004	0.001	[0.001, 0.007]
Direct effect (BMI → PEF%pred)	0.001	0.001	[−0.002, 0.003]
Indirect effect (BMI → HGS → PEF%pred)	0.003	0.001	[0.003, 0.004]
Fully standardized indirect effect	0.026	0.003	[0.020, 0.032]

Notes: This table presents effect sizes, standard errors, and 95% confidence intervals for the mediation analysis. The indirect effect quantifies the pathway BMI → HGS → PEF%pred. The fully standardized indirect effect is also reported. Standard errors and confidence intervals for the indirect effects were estimated using 10,000 bootstrap resamples (n = 8784).

### Sensitivity analysis of BMI and HGS on risk of impaired lung function

To evaluate the robustness of our findings, we repeated the logistic regression analyses using alternative cut-off values for impaired PEF%pred (ranging from 70% to 100%). Across all thresholds, the associations of both BMI and HGS with reduced PEF remained statistically significant and in the same direction (Table 6). Odds ratios for BMI ranged from 0.954 to 0.971 and for HGS from 0.955 to 0.967, with all 95% confidence intervals excluding 1. This suggests that the main findings are robust and not sensitive to the specific threshold used to define reduced PEF.

**Table 6:** Sensitivity analysis.

Model Type	PEF%pred Cutoff (%)	OR	95% CI	p-value	Sample size
Standard model	80				
BMI		0.970	0.945, 0.995	0.021	671/8784
HGS		0.961	0.952, 0.970	<0.001	671/8784
Sensitivity analysis 1	100				
BMI		0.962	0.945, 0.980	<0.001	1639/8784
HGS		0.967	0.960, 0.974	<0.001	1639/8784
Sensitivity analysis 2	90				
BMI		0.971	0.951, 0.992	0.007	1088/8784
HGS		0.967	0.959, 0.975	<0.001	1088/8784
Sensitivity analysis 3	70				
BMI		0.954	0.921, 0.987	0.007	371/8784
HGS		0.955	0.944, 0.967	<0.001	371/8784

Note: Odds Ratios (OR) and 95% Confidence Intervals (95% CI) were estimated using logistic regression. The dependent variable is reduced PEF (PEF%pred cutoff of 70%/80%/90%/100%). OR, Odds Ratio; CI, Confidence Interval. p-values < 0.05 are considered statistically significant.

## Discussion

To our knowledge, this study, based on data from 8784 middle-aged and elderly Chinese adults in the CHARLS cohort, provides the first comprehensive analysis of the interrelationships between BMI, HGS, and PEF in individuals without diagnosed lung disease. Our findings indicate that both low BMI and reduced HGS are independently associated with higher odds of reduced PEF. While HGS statistically mediated a portion of the association between BMI and PEF%pred, the absolute indirect effect was extremely small and not clinically meaningful. These results highlight the importance of maintaining muscle strength and nutritional status for pulmonary health in aging populations, but do not support interventions targeting muscle strength or weight solely for the purpose of improving PEF%pred based on the observed mediation effect. By characterizing the pathways linking body composition, muscle strength, and respiratory function, this study provides insights that may assist early identification of at-risk individuals and inform future research aimed at preventing pulmonary function decline in older adults.

### Interpretation of results and underlying mechanisms

In this study, both lower BMI and reduced HGS were associated with greater odds of reduced PEF, consistent with previous research showing associations between underweight status, sarcopenia, and impaired pulmonary function^12^^,^^20^^,^^21^ The mediation analysis identified that HGS serves as a statistically significant intermediary in the association between BMI and PEF%pred. Although this pathway technically accounts for a substantial portion of the total statistical association, the absolute change in PEF%pred specifically attributable to HGS was extremely small (unstandardized indirect effect = 0.003, 95% CI: 0.003–0.004). This effect size is far below any established threshold for a minimum clinically important difference, suggesting that the physiological impact of this mediation is negligible in practical terms. Thus, while the statistical mediation is robust, its clinical impact is negligible and should not be overinterpreted. The cross-sectional design also precludes inference about directionality or causality, and it is possible that reduced PEF could contribute to inactivity or muscle loss, or that other unmeasured factors influence all three variables.

Several mechanisms may help explain these associations. Lower BMI may reflect not only reduced fat mass but also diminished muscle mass, potentially compromising respiratory muscle strength^22^ Inadequate nutritional status could exacerbate muscle wasting and weaken respiratory muscles, which might contribute to lower expiratory flow^23^ Recent studies have emphasized the role of muscle dysfunction in predicting respiratory impairment in older adults, even after accounting for traditional risk factors^24^ In particular, sarcopenia has been recognized as a contributor to decreased pulmonary function, even in the absence of overt lung disease^25^

These findings also fit within emerging concepts of multisystem or “pan-organ” frailty, in which low HGS and BMI are considered markers of global physiological decline affecting multiple organ systems, including both the musculoskeletal and pulmonary systems^3^^,^^4^ The observed association between reduced PEF and heart disease in our cohort further supports this multisystem perspective^26^ Systemic inflammation, which may be more pronounced in states of low BMI, could also contribute through increased secretion of pro-inflammatory cytokines (e.g., IL-6, TNF-α) that promote muscle wasting and impair lung tissue^27^^,^^28^

The present results do not imply that interventions to increase BMI or muscle strength will necessarily improve pulmonary function; rather, they support the hypothesis that these factors are interrelated markers of overall health and function in older adults. The observed associations persisted after adjustment for several comorbidities, suggesting that diminished muscle mass and strength may be particularly important for pulmonary health in this population. Nevertheless, the findings should be interpreted in light of the study’s limitations regarding causality and confounding.

Our results also reinforce the value of muscle strength, as measured by HGS, as an indicator of overall functional status and respiratory health. Previous work in COPD populations has found that reduced grip strength is linked to more severe airflow limitation and poorer outcomes^12^^,^^29^ Here, the association between low HGS and reduced PEF was observed even in participants without diagnosed lung disease, supporting the potential utility of muscle assessment in a broad range of older adults.

### Comparison to existing literature

While prior studies have established the association between low BMI and reduced PEF, the underlying mechanisms have seldom been explored^20^ Our data add to this literature by suggesting that muscle strength may be a key intermediary, consistent with evidence that age-related declines in muscle mass and strength are major determinants of functional limitation, including pulmonary function^21^^,^^30^^,^^31^

Our findings also touch on the so-called “obesity paradox”, wherein overweight and obesity appear protective against certain adverse outcomes in older adults, including reduced PEF. However, the apparent protective effect of higher BMI should be interpreted cautiously^32^ Potential explanations include residual confounding (e.g., by comorbidity burden or unmeasured illness severity), survival bias, and the limitations of BMI as a measure of body composition. BMI does not distinguish between fat and muscle mass, and the observed associations do not imply that weight gain per se is beneficial. It is possible that greater muscle mass, rather than adiposity, underlies the protective association of higher BMI in some studies^33^ These interpretations are consistent with the extensive literature on the obesity paradox in respiratory and geriatric medicine^34^ Further research using more precise measures of body composition is needed.

### Strengths and implications

Strengths of this study include the use of a large, nationally representative sample, which enhances generalizability to Chinese middle-aged and elderly adults^16^^,^^19^ The application of mediation analysis and dose-response modeling provides a nuanced view of the interrelationships among BMI, HGS, and pulmonary function. To our knowledge, this is the first study to clarify the statistical mediating role of muscle strength in the association between BMI and PEF%pred in a population without diagnosed lung disease. However, given the negligible effect size, our findings do not support clinical recommendations for muscle-strengthening or weight-modification interventions solely for the purpose of improving PEF. Rather, they highlight the need for a holistic approach to maintaining health in older adults^14^^,^^18^^,^^35^

## Limitations

Several limitations should be acknowledged. First, our analysis was restricted to PEF as the only pulmonary function parameter available in the CHARLS dataset, limiting our ability to assess other important indices such as FEV_1_ or FVC, and precluding differentiation between obstructive and restrictive ventilatory patterns. Thus, “reduced PEF” should not be interpreted as a specific disease phenotype. Second, although we adjusted for multiple covariates, important potential confounders, including physical activity level, occupational exposures, and detailed smoking history (pack-years), were not available and could influence both muscle strength and lung function. Third, grip strength, while practical and widely used, may not fully capture overall muscle function compared to more comprehensive assessments. Fourth, BMI is a limited measure of body composition, not distinguishing between muscle and fat mass. Fifth, the cross-sectional nature of the study precludes conclusions about temporality or causality, and reverse or bidirectional relationships are possible (e.g., reduced PEF may contribute to inactivity and muscle loss). Residual confounding and survival bias may also influence the observed associations. Another limitation is that PEF relies on maximal voluntary effort. Individuals with lower handgrip strength might not complete qualified PEF tests, which could introduce measurement artifacts and confound the association between the two indicators.

### Future directions

Future research should employ longitudinal designs with repeated measures of muscle strength, lung function, and body composition (e.g., DEXA), as well as more comprehensive adjustment for confounders such as physical activity and smoking history. This would help clarify the temporal and potentially causal relationships among BMI, muscle strength, and pulmonary function in aging populations. In addition, studies incorporating inflammatory biomarkers and detailed frailty assessments may provide further insight into the multisystem mechanisms underlying respiratory impairment in older adults.

## Conclusion

In this large cross-sectional study of Chinese middle-aged and elderly adults without diagnosed lung disease, HGS was found to mediate the association between BMI and PEF. Lower BMI was linked to higher odds of reduced PEF, mainly through reduced muscle strength. While the cross-sectional design limits causal conclusions, these findings suggest that muscle strength plays a key role in respiratory health. Given the small mediated effect, further research is needed to explore muscle-strengthening interventions. Prospective studies with comprehensive lung function and body composition assessments are required to validate these associations and guide effective prevention strategies for respiratory health in older adults.

## Authors’ contributions

Haowei Jin: Conceptualization; Methodology; Software; Investigation; Writing-original Draft.

Sheng Zhou: Funding acquisition; Supervision.

Xiaonan Zhang: Visualization; Validation; Investigation; Supervision; Writing-review & editing.

## Funding

This work was supported by the National Natural Science Foundation of China under Grant 62475001.

## Data availability

The datasets generated and/or analyzed during the current study are available from the corresponding author upon reasonable request.

## Declaration of competing interest

The authors declare no conflicts of interest.
